# Supplementary material for: A 3D-Printed Integrated Handheld Biosensor for the Detection of Vibrio parahaemolyticus
Source: Foods. 2024 Jun 5;13(11):1775. doi: 10.3390/foods13111775 (PMC11171811; doi:10.3390/foods13111775)
Supplement: Supplementary file 1 [file foods-13-01775-s001.zip › supporting information-proof.pdf]

Supplementary information for

A 3D-printed Integrated Handheld Biosensor for the Detection of *Vibrio parahaemolyticus*

Yuancong Xu[1,2], Qian Zhang[2,3], Yunyi Li[3], Xiaoxu Pang[3], Nan Cheng[2,3*]

Table of Contents

Supplementary materials and methods

1. Reagents -- S2
2. Genomic DNA extraction-- S2
3. Primers and probes -- S3
4. Preparation of the lateral flow strip --- S3
5. Characterization techniques -- S3

Supplementary table

Table S1 --- S4
Table S2 --- S5
Table S3 --- S6

Supplementary figures

Figure S1 -- S7
Figure S2 -- S8
Figure S3 -- S9
Figure S4 -- S10
Figure S5 -- S11
Figure S6 -- S12

Supplementary materials and methods

1. Reagents

NaCl, Tris, HCl, HAc, NaOH, RNase, proteinase K, isopropanol, potassium acetate, ethanol, phenol, chloroform, chloroauric acid tetrahydrate ($HAuCl_4 \cdot 4H_2O$), trisodium citrate, bovine serum albumin (BSA), Tween 20, Triton X-100, streptavidin, sodium chloride-sodium citrate buffer (SSC, 20 concentrate, pH 7.0), and phosphate buffered saline (PBS, 0.01 M, pH 7.4) were purchased from Sigma Chemical Company (St. Louis, MO, USA). Filter bag (Bagfilter P400) was purchased from Interscience (Paris, France). Absorbent pads (CFSP001700), glass fiber sample pads (CFSP001700), nitrocellulose membranes (135 s), conjugation pads (GFCP000800), and backing cards (HF000 MC100) were purchased from Millipore (Bedford, MA, USA).

2. Genomic DNA extraction

The DNA samples used for RPA detection were extracted according to our previous method and device [36, 37] which improved on the Wizard magnetic DNA purification Kit.

(1) Dissect 100 mg fresh pork tissue and transfer into a 2 mL microcentrifuge tube. Add 500 μL lysis buffer A and 5 μL RNaseA, shake vigorously by hand for 10 s until well mixed.

(2) Add 250 μL lysis buffer B, shake vigorously for 10 s and let the sample stand at room temperature for 1 min.

(3) Add 750 μL precipitation solution and shake vigorously for 10 s.

(4) Transfer the solution into a new centrifuge tube while filtered through a 1 mm gauze.

(5) Add 50 μL resuspended MagneSil®PMP and shake vigorously for 5 s. (Note: MagneSil®PMP was mixed vigorously for 5 s before adding to the sample)

(6) Add 800 μL isopropanol and invert 10-15 times.

(7) Place the microcentrifuge tube to a magnetic separation device for 30 s, allowing the magnetic particles to completely clear from the solution. Discard the supernatant carefully.

(8) Add 250 μL lysis buffer B and invert 2-3 times.

(9) Place the microcentrifuge tube to a magnetic separation device for 30 s, allowing the magnetic particles to completely clear from the solution. Discard the supernatant carefully.

(10) Add 1 mL 70% ethanol and invert 2-3 times.

(11) Place the microcentrifuge tube to a magnetic separation device for 30 s, allowing the magnetic particles to completely clear from the solution. Discard the supernatant carefully.

(12) Repeat step 10 and 11 for two times. Clear the solution as possible.

(13) Remove the microcentrifuge tube from the magnetic separation device. Add 100 μL nuclease-free water to elute DNA. Invert 5 times and incubate at 37 °C (subsequent RPA temperature) for 1 min.

(14) Place the microcentrifuge tube to a magnetic separation device for 30 s until

all the magnetic particles are cleared from the solution. Transfer the supernatant containing purified DNA to a new collection microcentrifuge tube and store.

The DNA were measured and evaluated using a Nanodrop 1000 UV/Vis spectrophotometer (Nanodrop Technologies Inc., Wilmington, Delaware, USA). The DNA quality was analyzed by 1% (w/v) agarose gel electrophoresis.

3. Primers and probes

RPA primers to target the *tlh* gene (GeneBank: AB012596.1) of *V. parahaemolyticus*, were designed according to the Appendix of the TwistAmpTM reaction kit manuals. All probes were listed in the Table S2, including the ligation probe (LP), control line capture probe (CCP), test line capture probe (TCP) and AuNP-probe (AP). The 5'-terminus of CCP was modified with biotin (BIO) for immobilization to the control line. The 3'-terminus of TCP was also modified with BIO to prevent movement on the test line. The 5'-terminus of AP was modified with thiol group (SH) for immobilization to the AuNPs.

4. Preparation of the lateral flow strip

Lateral flow strips were prepared according to methods previously established in our laboratory [28]. The lateral flow strip was assembled with five parts, including the sample pad, conjugate pad, absorption pad, nitrocellulose membranes, and PVC backing. The sample pad and the conjugate pad were made from glass fiber, saturated with a buffer (containing 0.25% Triton X-100, 0.05 M Tris-HCl and 0.15 M NaCl, pH 8.0) for 30 min, dried at 37 °C for 2 h and stored in a dry environment. 20 μL of streptavidin (1 mg/mL) and 20 μL of CCP and TCP (100 μM) were mixed and incubated for 1 h, and then the mixture was sprayed on the nitrocellulose membrane (25 mm×30 cm) using a BioDot BioJet BJQ 3000 dispenser (Irvine, CA) at a speed of 1 μL/cm to form control line (CL) and test line (TL), respectively. The nitrocellulose membrane sprayed by the CL and TL was dried at 37 °C for 1 h and then stored at 4 °C. Then, the sample pad, conjugate pad, nitrocellulose membrane and absorption pad were assembled on a PVC backing (60 mm×30 cm) in a certain stacking order, leaving a 2 mm overlap width between each part to ensure the smooth flow of the solution onto the biosensor. Finally, a programmable slitter was used to cut the assembled lateral flow strip into finished products with widths of 1.5 mm. The lateral flow strips were stored under dry conditions at room temperature until use.

5. Characterization techniques

In this study, the extracted genomic DNA and RPA products were analyzed by 2% agarose gel electrophoresis. The fluorescence of CRISPR/Cas12a cleavage products was measured with 492 nm excitation and 518 nm emission. The photographs of fluorescence were taken by iPhone 12 in a dark room with 365 nm LED excitation. The transmission electron microscopy (TEM) was used to characterize the synthesized AuNPs. An ultraviolet visible (UV-Vis) spectrophotometer was used to characterize AuNPs and AuNP-AP.

Supplementary table

Table S1 Bacteria used in the study.

No.	Bacterias	Stains
1	*Vibrio parahaemolyticus*	ATCC 17802
2	*Salmonella spp.*	CGMCC 1.1552
3	*Listeria monocytogenes*	ATCC 19112
4	*Pseudomonas aeruginosa*	ATCC 47085
5	*Escherichia coli*	CICC 10899
6	*Staphyloccocus aureus*	CICC 10306
7	*Shigella sonnei*	CICC 21535
8	*Clostridium perfringens*	ATCC 10388
9	*Bacillus cereus*	ATCC 21928
10	*Enterobacter sakazakii*	ATCC 51329

Table S2. Primer sequence optimized for event-specific RPA reaction.

	Primer name	Sequence (5'to3')	Product length (bp)
RPA-CRISPR/Cas12a	VP-F1	CTCCCGCTTGCTTCTGCAGTTGCC GAAGAGCC	157
	VP-R1	CCCAATCGGTCGCTGGATCATCTT TCGAGTAGC	
	VP-F2	CACCTTGTTTACGCTTGAGTTTGG TTTGAATGAC	187
	VP-R2	CTCTTCTTGTGTTGAGTACTTAAA CTGAGGCGC	
	VP-F3	CATCACGTTGTTTGATACTCACGC CTTGTTCG	264
	VP-R3	TACTCGGCTAAGTTGTTGCTACTT TCTAGC	
	VP-crRNA	UAAUUUCUACUAAGUGUAGAU UUGCGUGCUGAUCACUUCAG	/
	cut probe	FAM-AGTACCGATAGATACAGAC-BHQ1	/
Lateral Flow Strip	CCP	BIO-ATACAGAC	/
	TCP	CTATCGGTACTATTTT-BIO	/
	AP	SH-TTTTTTTTGTCTGTAT	/
	LP	AGTACCGATAGATACAGAC	/

Table S3. Comparison of sensitivity between different methods.

Method	LOD	Detection time	On-site	References
3D-printed integrated handheld biosensor	4.9 CFU/mL	23 min	Yes	this method
SERS	10^5 CFU/mL	——	No	[7]
LAMP	53 CFU/mL	22-40 min	No	[41]
Real-time RPA	10^2 copies/reaction	5–12 min	No	[42]
Colorimetric LAMP	1.0 CFU/mL	30 min	Yes	[43]
RPA-LF	3×10^2 CFU	15 min	Yes	[44]
QDs based immunofluorescence	10^2 CFU/mL	150 min	No	[8]
Aptamer-based SPR biosensor	——	——	No	[10]
MOF-colorimetric biosensor	10^2 CFU/mL	1.5 h	Yes	[11]
CRISPR-colorimetric biosensor	4.9×10^2 CFU/mL	185 min	Yes	[21]
PCR-CRISPR biosensor	1.02×10^2 copies/μL	85 min	No	[45]

Supplementary figure

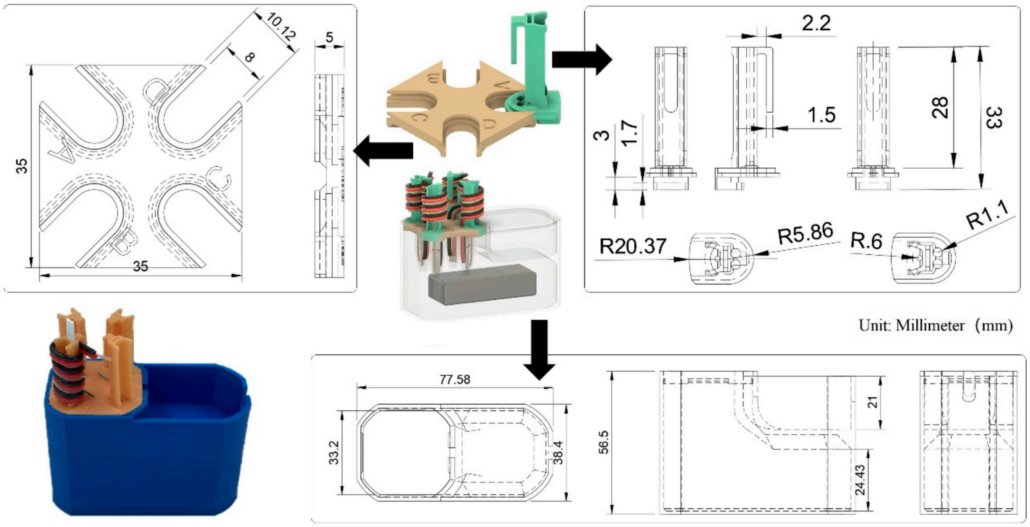

Figure S1. The diagram of 3D printed handheld device with all the dimensions labeled.

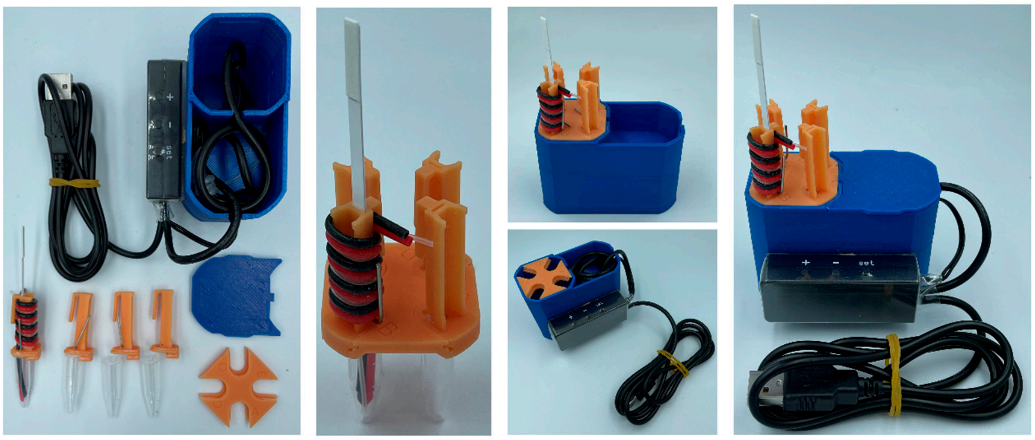

Figure S2. The real object of 3D printed handheld device.

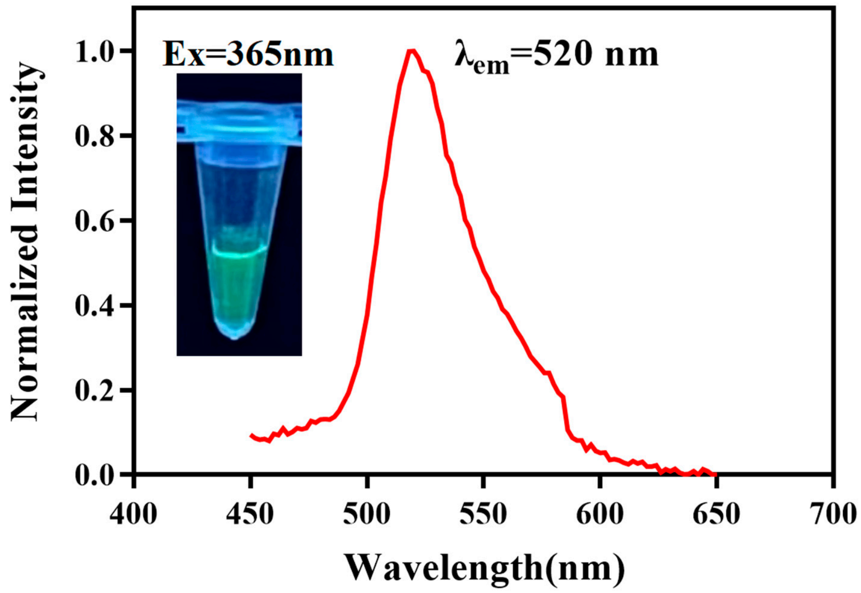

Figure S3. A fluorescence image and an emission spectrum of FAM with excitation at 365 nm.

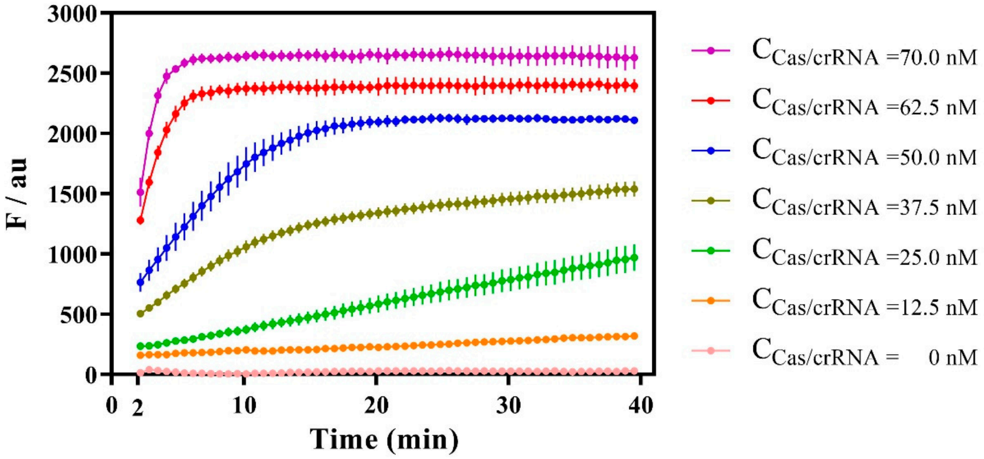

Figure S4. Fluorescence curve of different Cas12a/crRNA concentration (RPA time 10 min, cut probe 200 nM).

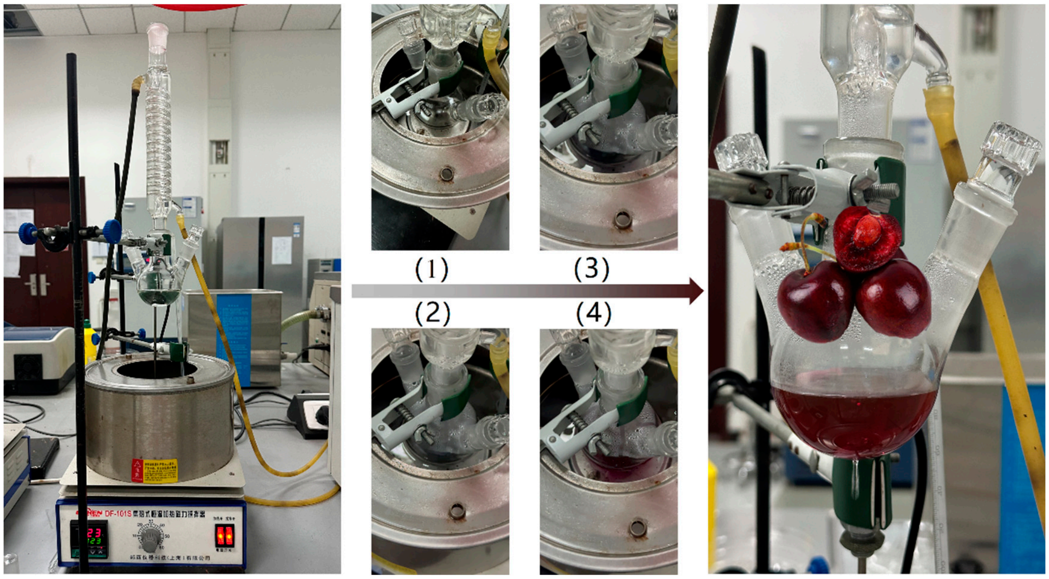

Figure S5. Process images related to AuNP preparation.

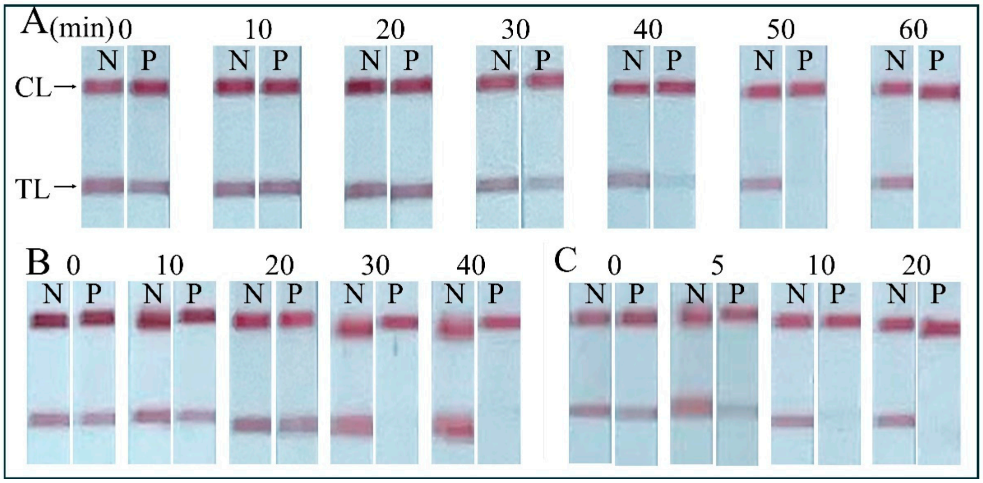

Figure S6. Optimization of the LP concentration with different CRISPR cut time. (A) 600 nM; (B) 300 nM; (C)100 nM.
